# A Study on Factors Affecting the Degradation of Magnesium and a Magnesium-Yttrium Alloy for Biomedical Applications

**DOI:** 10.1371/journal.pone.0065603

**Published:** 2013-06-14

**Authors:** Ian Johnson, Huinan Liu

**Affiliations:** Department of Bioengineering, University of California Riverside, Riverside, California, United States of America; University of Akron, United States of America

## Abstract

Controlling degradation of magnesium or its alloys in physiological saline solutions is essential for their potential applications in clinically viable implants. Rapid degradation of magnesium-based materials reduces the mechanical properties of implants prematurely and severely increases alkalinity of the local environment. Therefore, the objective of this study is to investigate the effects of three interactive factors on magnesium degradation, specifically, the addition of yttrium to form a magnesium-yttrium alloy versus pure magnesium, the metallic versus oxide surfaces, and the presence versus absence of physiological salt ions in the immersion solution. In the immersion solution of phosphate buffered saline (PBS), the magnesium-yttrium alloy with metallic surface degraded the slowest, followed by pure magnesium with metallic or oxide surfaces, and the magnesium-yttrium alloy with oxide surface degraded the fastest. However, in deionized (DI) water, the degradation rate showed a different trend. Specifically, pure magnesium with metallic or oxide surfaces degraded the slowest, followed by the magnesium-yttrium alloy with oxide surface, and the magnesium-yttrium alloy with metallic surface degraded the fastest. Interestingly, only magnesium-yttrium alloy with metallic surface degraded slower in PBS than in DI water, while all the other samples degraded faster in PBS than in DI water. Clearly, the results showed that the alloy composition, presence or absence of surface oxide layer, and presence or absence of physiological salt ions in the immersion solution all influenced the degradation rate and mode. Moreover, these three factors showed statistically significant interactions. This study revealed the complex interrelationships among these factors and their respective contributions to degradation for the first time. The results of this study not only improved our understanding of magnesium degradation in physiological environment, but also presented the key factors to consider in order to satisfy the degradation requirements for next-generation biodegradable implants and devices.

## Introduction

Magnesium alloys possess many advantageous properties over current materials used for biomedical implants. Magnesium is biodegradable and its degradation (or corrosion) products can be effectively metabolized and excreted through the kidney in healthy adults [1]. The mechanical strength and elastic modulus of magnesium alloys are similar to cortical bone, and thus magnesium alloys can be used for load-bearing implants with minimal stress shielding [2]. Magnesium alloys also enhance bone growth as compared with current implant materials, i.e., titanium alloys or stainless steels [3], [4].

Despite the many desirable properties of magnesium for medical implant and device applications, the rapid degradation of magnesium *in vivo* remains a critical challenge [5]. Many interacting factors affect magnesium degradation, such as bulk composition of magnesium alloys, microstructure and composition at the surface of the magnesium alloy, and the composition of surrounding fluids. Understanding the interactions between these factors is as important as recognizing the role of each individual factor on magnesium degradation. For example, one method of controlling magnesium degradation is to add certain alloying elements into the magnesium and Yttrium (Y) is often added to magnesium alloys to increase material strength [6], [7], ductility [8], and degradation (or corrosion) resistance [9], [10], [11], [12], [13]. However, Y exhibits both degradation inhibiting and degradation promoting activities in magnesium alloys, depending on other factors [14]. Yttrium oxide (Y_2_O_3_) accumulates in the degradation layer when Y migrates to the metal surface and is oxidized [15], [16], [17]. When a stable degradation layer is present, yttrium oxide in the degradation layer can slow down magnesium degradation by inhibiting cathodic reactions [17]. When the degradation layer is not stable, Y can promote microgalvanic corrosion by making the β phase more cathodic [15]. The initial alloy surface plays a critical role in determining the overall effects of Y on the degradation of magnesium alloys [14]. In addition, certain alloy compositions and surface properties that improve corrosion resistance in one environment may accelerate degradation in another environment. Therefore, it is important to elucidate the interactions among the factors influencing magnesium alloy degradation in order to tailor magnesium alloys more effectively for their intended applications at various anatomical locations *in vivo*, thus achieving desirable life span for magnesium-based biodegradable implants.

Physiological salt ions in body fluids can aggressively attack magnesium and accelerate its degradation. Rapid degradation can result in mechanical failure of implants before the healing tissues regain their mechanical strength. Magnesium degradation also produces hydroxide ions and hydrogen gas. The hydroxide ions can significantly increase the local pH, which may have adverse effects on local cell functions. The hydrogen gas evolution can lead to the formation of gas cavities in the tissue [1]. Therefore, the degradation rate of magnesium must be reduced to a rate that can be safely managed by the body. Magnesium degradation in physiological fluids mostly occurs through reaction 1 below [18], [19], [20]. In aqueous environments, a degradation layer composed of Mg(OH)_2_ forms on the surface of magnesium through reaction 1b. The degradation layer only provides limited protection to magnesium from subsequent degradation due to its loose and porous microstructure.
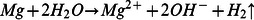
(1a)

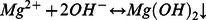
(1b)


Abundant chloride ions (Cl^−^) in physiological fluids undermine the degradation layer on magnesium. Microgalvanic coupling causes the α phase of magnesium alloys to degrade more rapidly than the β phase [21]. Cl^−^ is electrochemically transported to the more anodic α phase where it is adsorbed [22]. The adsorption of Cl^−^ to magnesium was greater than the adsorption of other halide ions to magnesium, which has been demonstrated by the greater surface capacitance of magnesium after exposure to Cl^−^ [21]. Adsorption of halide ions onto magnesium is competitive [23]. Adsorbed Cl^−^ reduces the corrosion potential of magnesium and converts Mg(OH)_2_ into the much more soluble MgCl_2_ [24], [25]. The magnitude by which halide ions reduce the corrosion potential of magnesium is proportional to the solubility of the salts that halide ions form with magnesium [26]. The high solubility of MgCl_2_ drives dissolution of magnesium alloys. Cl^−^ is extremely corrosive to magnesium alloys because of the combination of electrochemical transportation of Cl^−^ to anodic regions, efficient and competitive adsorption of Cl^−^ onto the surface of magnesium alloys, and the high solubility of the products that Cl^−^ forms with magnesium. Because of these combined factors, dissolution of the degradation layer exposes the underlying metallic phase, thus making it prone to further degradation. Phosphates can increase the density of the degradation layer and increase its resistance to Cl^−^ attack [27]. Carbonates can increase the stability of the degradation layer and inhibit pitting degradation processes [28]. Many other constituents or ions in physiological fluids may also influence magnesium degradation [29].

**Figure 1:**
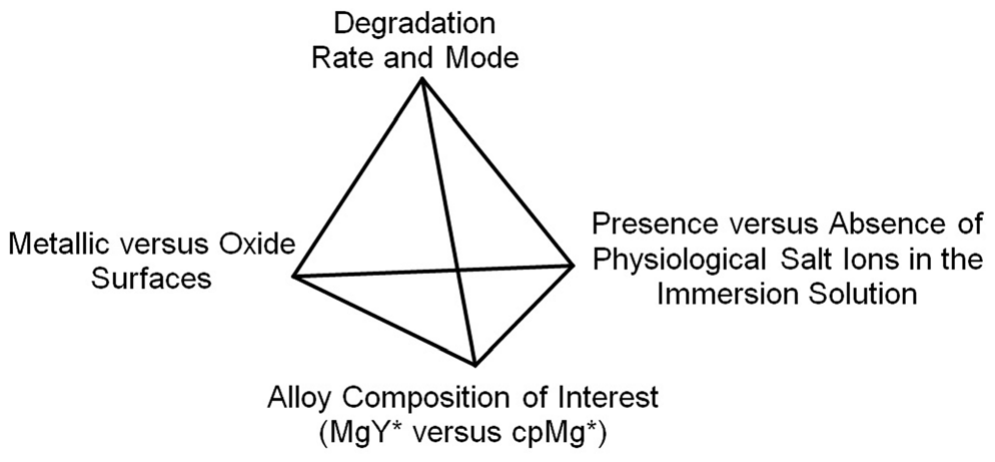
Three key interacting factors influence degradation rate and mode of magnesium. MgY* refer to Mg-Y alloys with either metallic (MgY) or oxide (MgY_O) surfaces. cpMg* refers to commercially pure Mg with either metallic (cpMg) or oxide (cpMg_O) surfaces.

The objective of this study was to investigate the roles of three key factors and their interactions in determining magnesium degradation: the presence or absence of yttrium in magnesium alloys, the presence or absence of surface oxides, and the presence or absence of physiological ions in the immersion fluid (Figure 1). Specifically, the degradation of magnesium-4wt.% yttrium alloy was studied in comparison with 99.9% commercially pure (cp) magnesium. Both magnesium-yttrium alloy and pure magnesium samples were studied in two kinds of surface conditions, i.e., metallic versus oxide surfaces. A phosphate buffered saline (PBS) solution containing physiological salt ions and deionized (DI) water were used as immersion solutions.

## Materials and Methods

### Preparation of pure magnesium and magnesium-yttrium alloy samples

Commercially pure magnesium foil (Goodfellow Corporation, as-rolled, 99.9% purity) with a thickness of 250 µm was used as a control in this study. The as-rolled magnesium foil had a thermal oxide layer on its surface and was called cpMg_O in this study with “cp” indicating commercially pure and “O” indicating the presence of oxides on the surface. Some of the cpMg_O samples were grinded using 600, 800, and 1200 grit silicon carbide abrasive papers (PACE Technologies) to remove the oxide layer on the surface, and were referred to as cpMg in this study. The term cpMg* was used to refer to both cpMg_O and cpMg in this manuscript.

Magnesium-4 wt.% yttrium alloy was prepared by melting magnesium with 4wt.% yttrium in an argon protected environment and casting as an ingot. The as-cast magnesium-yttrium alloy ingot was cut into 250 µm thick discs using a wire electric discharge machine (AgieCharmilles, Agiecut 200 VHP). The as-produced alloy discs had a thermal oxide layer on their surface and were called MgY_O in this study. Some of the MgY_O samples were grinded using 600, 800, and 1200 grit silicon carbide abrasive papers to remove the oxide layer on the surface, and were referred to as MgY in this study. MgY* was used to refer to both MgY_O and MgY in this manuscript.

All of the cpMg* and MgY* samples in this study were cut into dimensions of 10 mm×10 mm, cleaned in isopropanol (Sigma-Aldrich, CAS number 67-63-0), and weighed. Both sides of the samples were disinfected under ultraviolet (UV) radiation for at least 8 hours before degradation experiments.

### Degradation of pure magnesium and magnesium-yttrium alloy in immersion solution

Degradation of pure magnesium and the magnesium-yttrium alloy was investigated by the immersion method. Briefly, both cpMg* and MgY* samples were immersed in two different immersion solutions for comparison: deionized (DI) water and phosphate buffered saline (PBS). DI water was produced by a Millipore Milli-Q® Biocel System and used as a control. PBS was prepared by dissolving 8 g NaCl, 0.2 g KCl, 1.5 g Na_2_HPO_4_, and 0.2 g KH_2_PO_4_ in 1000 ml of DI water and adjusting the pH to 7.4 (all chemicals from Sigma). PBS was chosen as one of the immersion solutions in order to determine the effects of aggressive physiological ions (e.g. Cl^−^) on magnesium degradation. Both PBS and DI water were sterilized in an autoclave. Immersion occurred under standard cell culture conditions (a 37°C, 5% CO_2_/95% air, humidified, sterile environment) without stirring. Each sample was immersed in 3 mL of solution.

All cpMg* and MgY* samples were incubated in DI water and PBS according to the prescribed sequential time points. The incubation time was shorter (1 hour) at the beginning of the degradation experiment to provide a higher time resolution. A higher time resolution was necessary to track the initial rapid changes of sample mass and pH of immersion solution. Furthermore, the initial period of degradation plays a critical role on the fate of the surrounding cells. The alloy surface microstructure and local pH both have profound effects on cell survival and functions during the early stage of implantation. After 3 days of immersion, the incubation time was increased to 48 hours (2 days) to mimic normal physiological conditions. When the prescribed incubation time ended, the samples were removed from their immersion solution and dried in a 37°C isotemp oven for 12 hours, or until the sample reached a constant mass. Degradation products that precipitated on the surface of the cpMg* and MgY* samples were left intact, while soluble degradation products remained in the immersion solution. The pH meter was first calibrated with known standards, and then used to measure the pH of the immersion solution at the end of every prescribed incubation time. The samples were dried, weighed, photographed, disinfected under UV radiation, and then placed in fresh immersion solution for the next incubation time. The same procedure was repeated for each prescribed incubation cycle. When the sample mass was reduced to less than 3 mg, they became too small to handle and thus were considered as completely degraded at the next time point. The mass of the samples after each incubation time (M_i_) was divided by its initial mass (M_0_) to obtain the normalized mass change (M_i_/M_0_). The degradation tests were performed in triplicate for each sample type.

### Statistical analysis of degradation data

The three factors that control the dependent variable (i.e., sample degradation) were alloy composition (Mg or MgY), sample surface (metallic or oxide), and immersion media (DI water or PBS). Three-way factorial ANOVA was used to analyze the effects of these factors on the sample degradation, mainly the sample mass change during degradation. The Shapiro-Wilk test was used to verify that the data had a normal distribution. The Bartlett test was used to verify that the different sample groups had homogeneous variance. A significance level of α = 0.05 was used for all statistical tests. Two-way interaction plots were generated to illustrate the interactions between all possible combinations of two factors. All the statistical tests were performed using R.

### Surface characterization of magnesium and magnesium-yttrium alloy

The cpMg* and MgY* samples were disinfected under UV radiation, and then incubated in DI water and PBS under standard cell culture conditions (37°C, 5% CO_2_/95% air, humidified sterile environment) for 24 hours. After that, the samples were taken out of the immersion solution, and dried in a vacuum oven at room temperature for 2 days. The surfaces of cpMg* and MgY* samples were characterized before and after 24-hour degradation using a field emission scanning electron microscope (FESEM; Philips XL-30). Energy dispersive X-ray spectroscopy (EDS) analysis was performed at an accelerating voltage of 15 kV and a magnification of 2500× to determine elemental composition at the sample surface. Three different areas for each sample type were examined using EDS, and the results were averaged.

## Results

### Degradation of the cpMg* and MgY* samples in DI water

**Figure 2:**
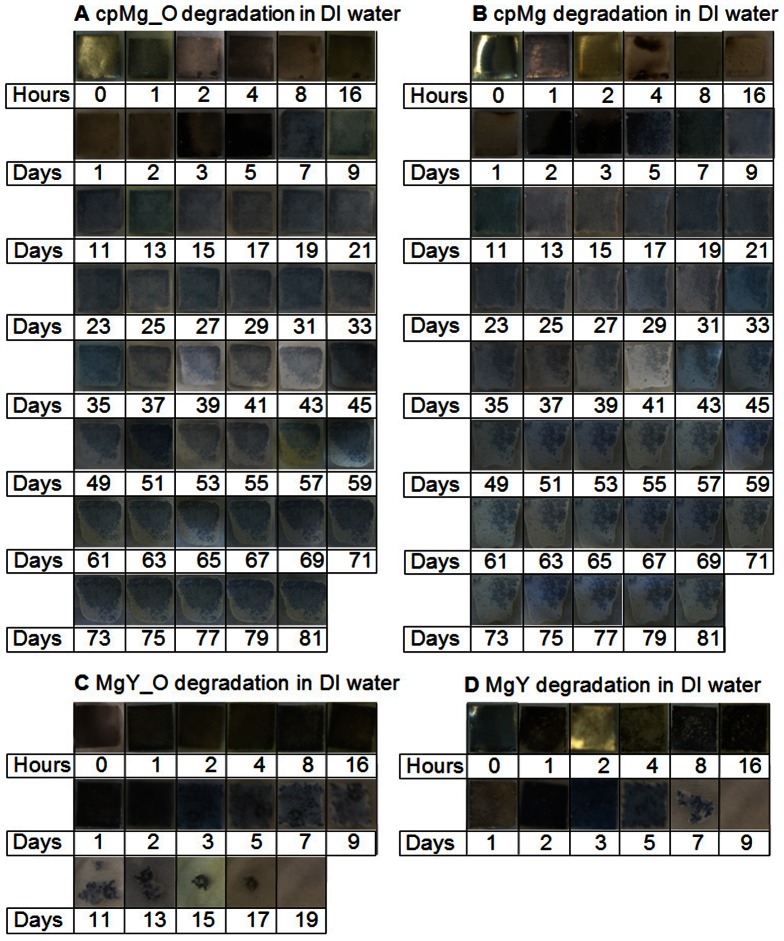
Photographs of the cpMg* and MgY* samples after incubation in DI water under standard cell culture conditions for the prescribed time points. The sample appearance changed with time, indicating different degradation rate and mode.

**Figure 3:**
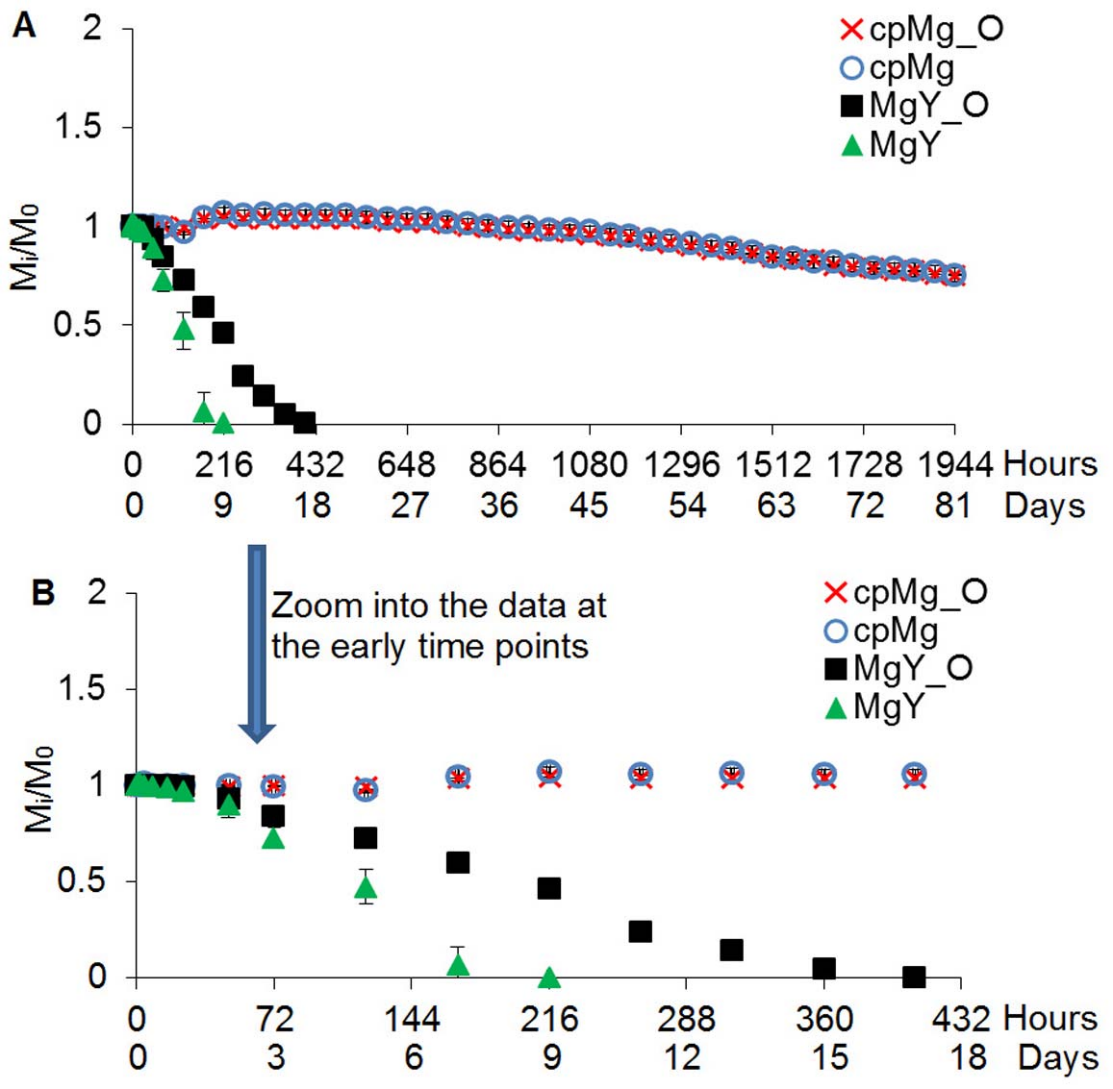
The mass change of the cpMg* and MgY* samples during degradation in DI water. M_i_ was the mass after the prescribed incubation time (i) and M_0_ was the initial mass. (A) Sample mass change at all time points. (B) Zoom into sample mass change at the early time points for clear view. Values are average ± standard deviation; N = 3.

**Figure 4:**
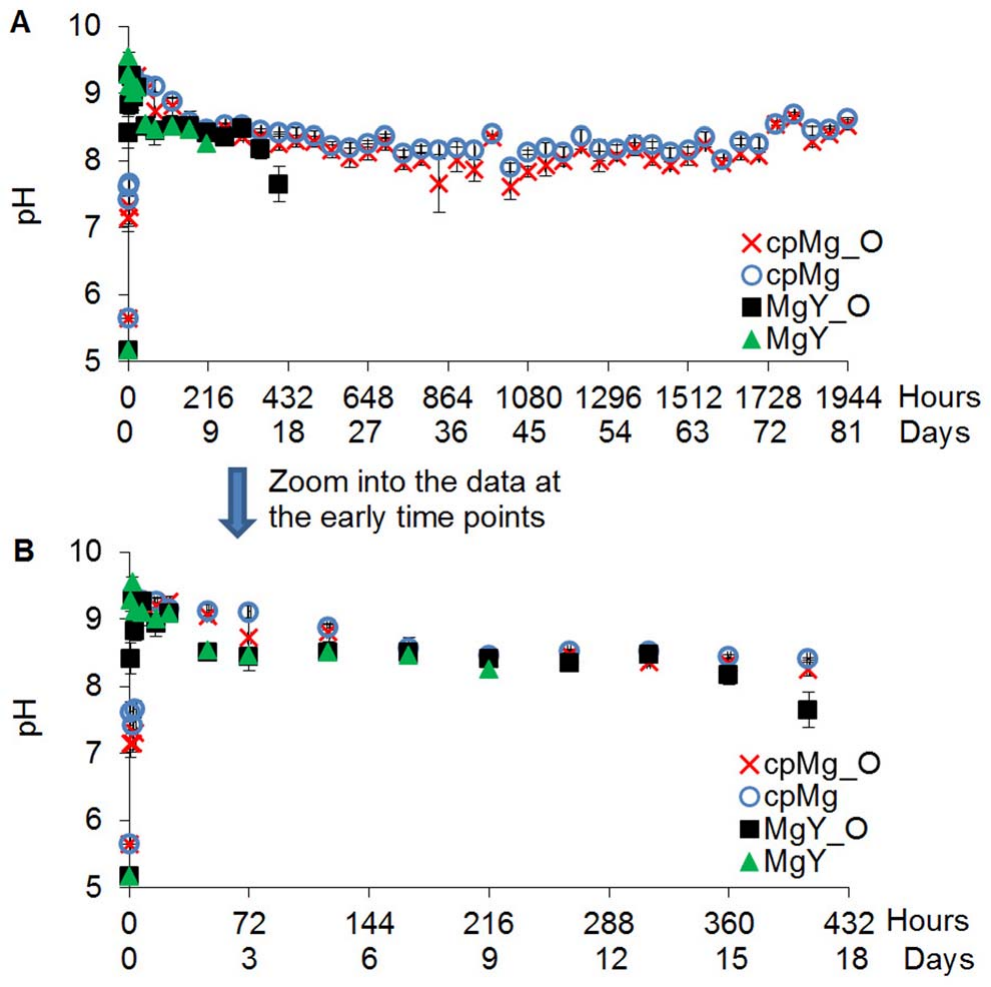
The pH change of the DI water containing the cpMg* and MgY* samples over the prescribed incubation time. (A) DI water pH at all time points. (B) Zoom into DI water pH at the early time points for clear view. Values are average ± standard deviation; N = 3.

The degradation results of cpMg* and MgY* samples in DI water were summarized as time-lapse photographs in Figure 2, sample mass change in Figure 3, and pH change of the DI water after sample immersion in Figure 4.

#### Degradation appearance in DI water

The surface of cpMg_O (Figure 2A) did not show significant change until the 3rd day of degradation in DI water. Dark-colored degradation products appeared on one side of the sample at the 3rd day and progressed across the entire surface by the 5th day. The degradation layer appeared gray and relatively homogeneous to visual inspection after the 5th day. Although the degradation layer covered the entire cpMg_O surface by the 5th day, the change of sample size was small. The degradation of samples initiated from the edges that slowly migrated inward while leaving behind a smooth contour. The surface of cpMg (Figure 2B) did not show significant change until the 2nd days of degradation in DI water. Dark-colored degradation products appeared on one side of the sample and then progressed across the entire surface by the 3rd day. The cpMg degradation seemed very similar as that observed on cpMg_O in DI water. The samples started to degrade from the edge and migrate inward. In contrast, the surface of MgY_O (Figure 2C) turned dark after only 1 hour of incubation in DI water. Localized gray degradation products gradually accumulated on the sample surface until the entire surface became dark gray by the 3rd day. As the degradation was not homogeneous, MgY_O shed fragments and lost structural integrity by the day 11 and completely degraded after 19 days. The surface of MgY (Figure 2D) appeared very similar as MgY_O. Most of the visible degradation of MgY occurred between 5 and 7 days, and MgY completely degraded after 9 days. MgY degraded much more rapidly than any other sample types in DI water.

#### Change of mass and pH of DI water

Figure 3 shows the mass change of the samples in DI water. The mass of cpMg_O samples was constant for the first 3 days of incubation in DI water, followed by slight mass loss between 3 and 5 days, slight mass gain between 5 and 9 days, and then slow yet continuing mass loss. The cpMg_O and cpMg samples had very similar mass loss at each time point. However, MgY* degraded much more rapidly than cpMg* in DI water. MgY* had constant mass between 0 and 16 hours of incubation in DI water, followed by rapid mass loss. MgY had even more rapid mass loss than that of MgY_O and the entire MgY sample was gone after 9 days.

Figure 4 shows the pH change of DI water after sample immersion. Degradation of cpMg_O caused a rapid pH increase in DI water to 9.2 during the first 24 hours, and then the pH gradually decreased. Between 9 and 29 days, the pH of DI water stabilized in the range of 8.0 to 8.4. The cpMg degradation resulted in very similar pH change as cpMg_O, with a few exceptions at the early stage and at the end. The DI water containing cpMg was slightly more alkaline than cpMg_O at some time points. The DI water containing MgY_O reached its maximum pH of 9.29 during the first 2 hours of incubation, and the pH reached a plateau at 8.4 afterwards until 13 days of incubation. After 13 days, the pH started to decrease and reached 7.64 at 18 days due to significant reduction of sample size. MgY degradation resulted in a similar trend in pH change as observed for MgY_O, except slightly more alkaline during the first 4 hours.

### Degradation of the cpMg* and MgY* samples in PBS

**Figure 5:**
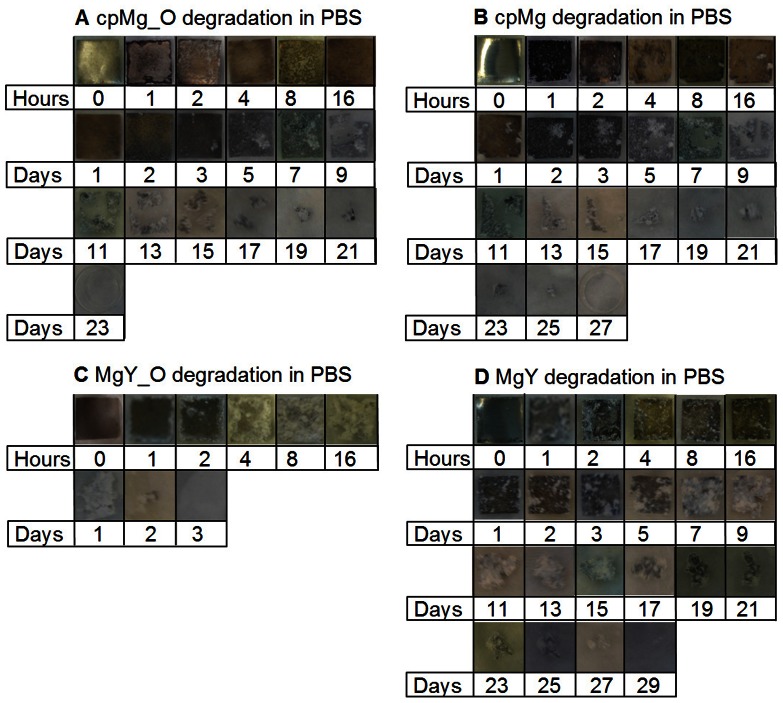
Photographs of the cpMg* and MgY* samples after incubation in PBS under standard cell culture conditions for the prescribed time points. The sample appearance changed with time, indicating different degradation rate and mode.

**Figure 6:**
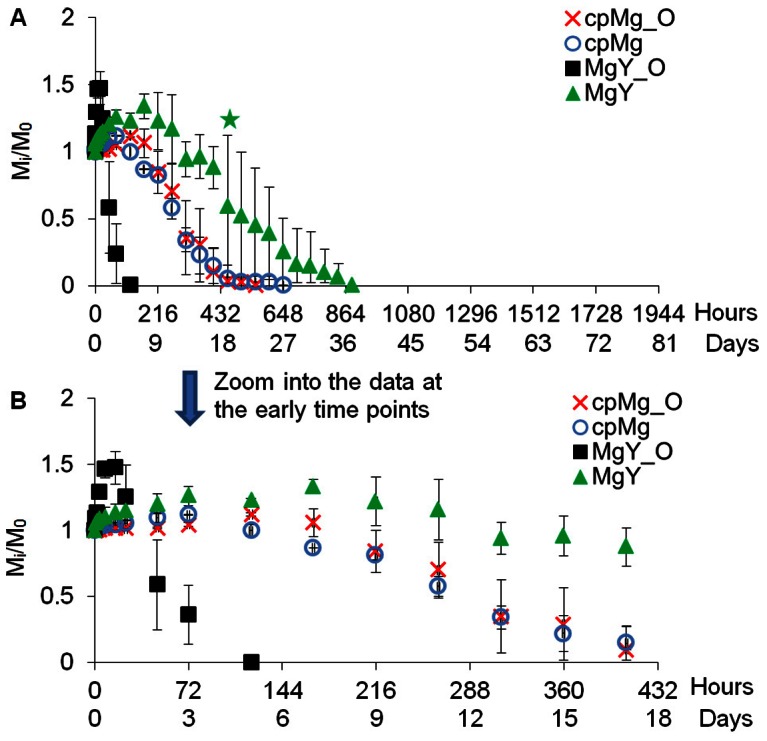
The mass change of the cpMg* and MgY* samples during degradation in PBS. M_i_ was the mass after the prescribed incubation time (i) and M_0_ was the initial mass. (A) Sample mass change at all time points. (B) Zoom into sample mass change at the early time points for clear view. The green star above the error bar of MgY mass change at 456 hr indicates that one of the triplicate samples completely degraded (i.e., reached 0 mass), causing a greater standard deviation. Values are average ± standard deviation; N = 3.

**Figure 7:**
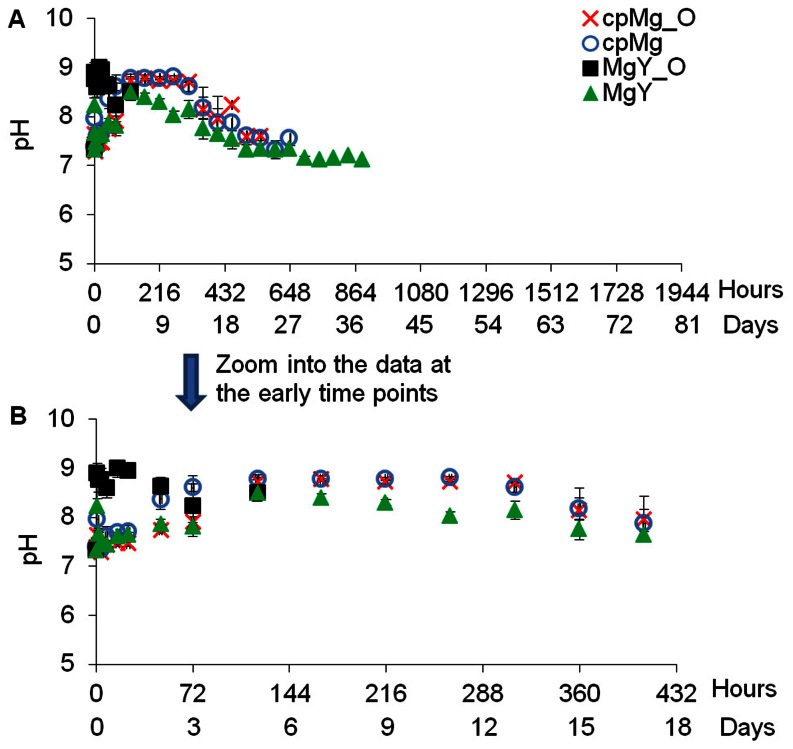
The pH change of the PBS solution containing the cpMg* and MgY* samples over the prescribed incubation time. (A) PBS pH at all time points. (B) Zoom into PBS pH at the early time points for clear view. Values are average ± standard deviation; N = 3.

The degradation results of cpMg* and MgY* samples in PBS were summarized as time-lapse photographs in Figure 5, sample mass change in Figure 6, and pH change of the PBS after sample immersion in Figure 7.

#### Degradation appearance in PBS

Rough and inhomogeneous gray degradation products accumulated on the surface of cpMg_O after immersion in PBS for only 1 hour (Figure 5A). Once the degradation products covered the entire surface after 3 days, accumulation of white degradation products appeared near the center of the sample. The cpMg_O samples fragmented in the center at day 9, the large fragments continued to degrade and eventually dissolved after 23 days. In Figure 5B, similar degradation products accumulated on the surface of cpMg and spread at a similar rate. The cpMg samples fragmented near the center at day 5 and the remaining fragments continued to degrade until completely dissolved after 27 days. In Figure 5C, white degradation products accumulated at the edges of MgY_O samples after immersion in PBS for 1 hour. The degradation layer was rough, porous, and heterogeneous, and migrated inward from the edge until it covered the entire surface. MgY_O continuously shed fragments from its edges after 16 hours and completely degraded after 3 days. As shown in Figure 5D, localized white degradation products appeared on the surface of MgY after 1 hour of incubation in PBS and spread over entire surface in 2 days. The MgY samples started to release fragments from its edges after day 5 and completely degraded after 29 days.

#### Change of mass and pH of PBS

Figure 6 shows the mass change of the samples in PBS. The mass of cpMg_O was constant for the first 2 days, slightly increased until day 5, and rapidly decreased afterwards. The mass change of cpMg over time was similar as that of cpMg_O in PBS with a few exceptions. For example, cpMg reached its peak mass in a shorter time (i.e., 3 days) and lasted slightly longer than cpMg_O. Interestingly, MgY_O immersed in PBS reached the peak mass the first, showed the greatest mass loss in the shortest incubation time among all the samples tested. The MgY samples gained more mass than that of cpMg* samples in PBS, but took longer to reach the peak mass. After reaching the peak mass, the sample mass started to decrease gradually. It is interesting to point out that the mass change of all samples had much greater deviation than their respective mass change in DI water, as indicated by bigger error bars. Inhomogeneous sample degradation in PBS may have contributed to the large variances.

Figure 7 shows the pH change of PBS after sample immersion. The pH of PBS immersed with cpMg_O reached the maximum of 8.73 after 5 days of incubation, remained at that plateau until 13 days, and then slowly decreased to 7.62 at the end time point. The PBS containing cpMg had the same pH plateau as cpMg_O, although the plateau was reached slightly earlier. The pH of the PBS containing MgY_O rapidly increased to 8.90 during the first hour, and then slightly decreased to around 8.40 until the samples degraded entirely. The pH of the PBS containing MgY rapidly increased to 8.2 during the first hour of incubation, reached its peak value of 8.49 at 5 days, and then continually decreased to 7.19 at the end.

### Statistical analysis of mass loss during degradation

There were significant interactions among alloy composition, sample surface type, and immersion media, as demonstrated through three-way factorial ANOVA analysis. The dependent variable should be a direct indicator of the sample degradation. The pH data did not have homogenous variance, and thus was not suitable as the dependent variable in the statistical analysis. The data on sample mass change did not meet the criteria of normal distribution, either. Therefore, the log (sample lifetime) was introduced as the dependent variable because it had normal distribution and homogenous variance, which met the criteria for three-way factorial ANOVA. The sample lifetime was defined as the time point when the sample was considered completely degraded or its residual mass was less than 3 mg. The lifetime of the samples that never fully degraded (i.e., cpMg* in DI water) by the end of the degradation study (i.e., 1944 hours) were calculated based on their maximum degradation rate recorded in the study.

Alloy composition, sample surface type, and immersion media all had statistically significant effects on sample lifetime, as shown in Table 1; with values of *p* = 1.10×10^−13^, *p* = 4.37×10^−2^, and *p* = 2.87×10^−11^ respectively. Furthermore, statistically significant two-way interactions were observed between alloy composition and sample surface (*p* = 2.95×10^−4^), alloy composition and immersion media (*p* = 4.45×10^−11^), and sample surface and immersion media (*p* = 3.47×10^−8^). A statistically significant three way interaction was also observed between alloy composition, sample surface, and immersion media (*p* = 1.03×10^−5^).

**Table 1:** Statistical significance of the three factors (alloy composition, sample surface type, immersion media) on sample lifetime and their interactions.

Three-way Factorial ANOVA	Alloy	Surface	Media	Alloy- Surface Interaction	Alloy- Media Interaction	Surface- Media Interaction	Alloy- Surface- Media Interaction
*p* value	1.10×10^−13^	4.37×10^−2^	2.87×10^−11^	2.95×10^−4^	4.45×10^−11^	3.47×10^−8^	1.03×10^−5^

The *p* values were calculated using three-way factorial ANOVA. All *p* values were <0.05, indicating statistical significance.

**Figure 8:**
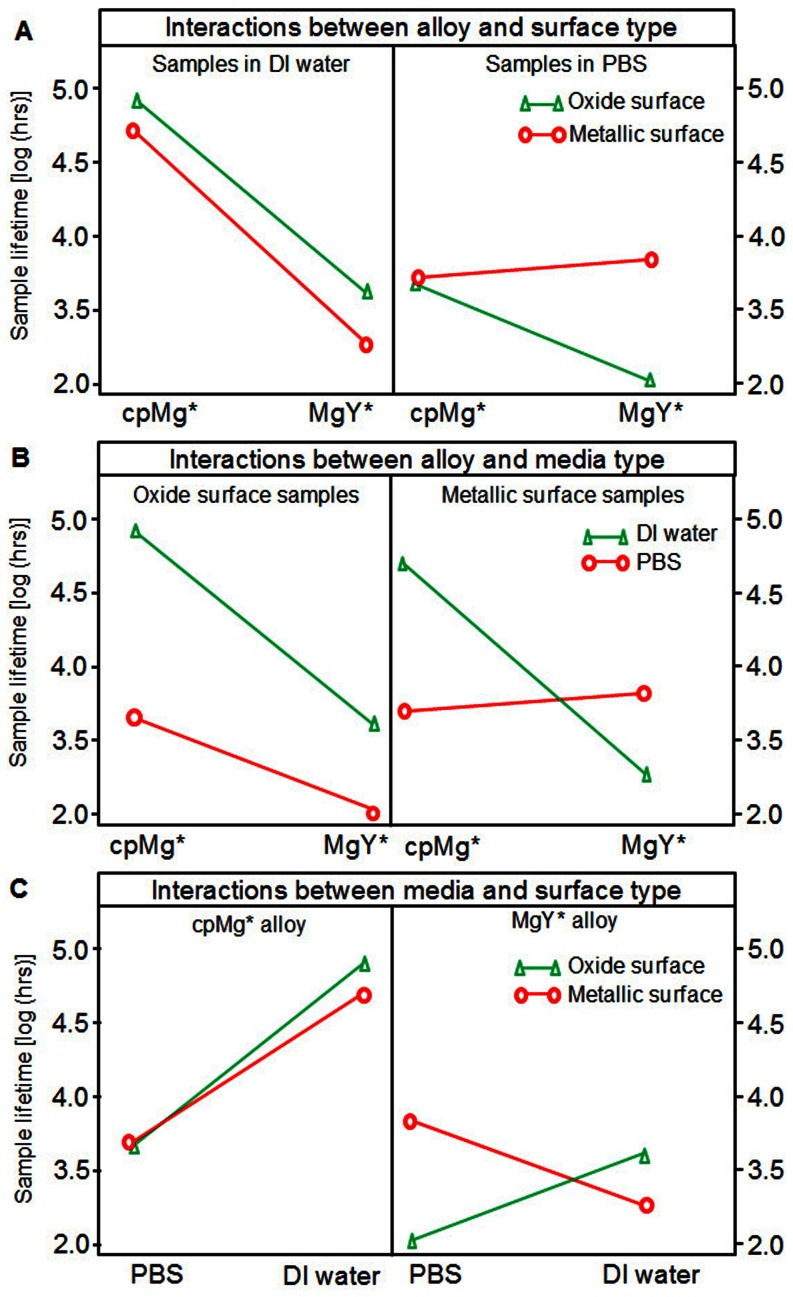
Interactions between three key factors that influence sample lifetime. Interaction plots demonstrated the two-way interactions between factors. Two separate interaction plots with different values of the third factor were placed side by side for comparison. The values of the third factor were shown directly above each graph. (A) Alloy and surface type with media type as the third factor. (B) Alloy and media type with surface type as the third factor. (C) Media and surface type with alloy as the third factor.

Two-way interaction plots graphically demonstrated the relationships among the three factors and their combined effects on the log (sample lifetime, hrs), as shown in Figure 8. The different values for one factor are presented along the X axis, while the Y axis represents the log (sample lifetime). Two different lines in each plot present the different values for the second factor. The relationships between these two factors are further affected by the third factor and are thus plotted side by side for comparison. Figure 8A had two almost parallel lines with negative slopes for the left plot, and a “<” shape with one positive and one negative slope for the right plot. Figure 8B had two almost parallel lines of a small angle with negative slopes for the left plot, and an intersected “X” shape with one positive and one negative slope for the right plot. Figure 8C had a “<” shape with two positive slopes for the left plot, and an intersected “X” shape with one positive and one negative slope for the right plot. The slopes of these lines, as well as the angles and types of intersections revealed the interactions among three factors. Interaction plots with two intersected lines showed substantial interaction between the two factors, while those with almost parallel lines showed no to little interaction between the two factors. A “<” shape showed consistent interactions between the two factors, while an “X” shape demonstrated that the interactions between the two factors had opposite effects at different values of the third factor.

### Surface microstructure and composition of cpMg* and MgY* samples

**Figure 9:**
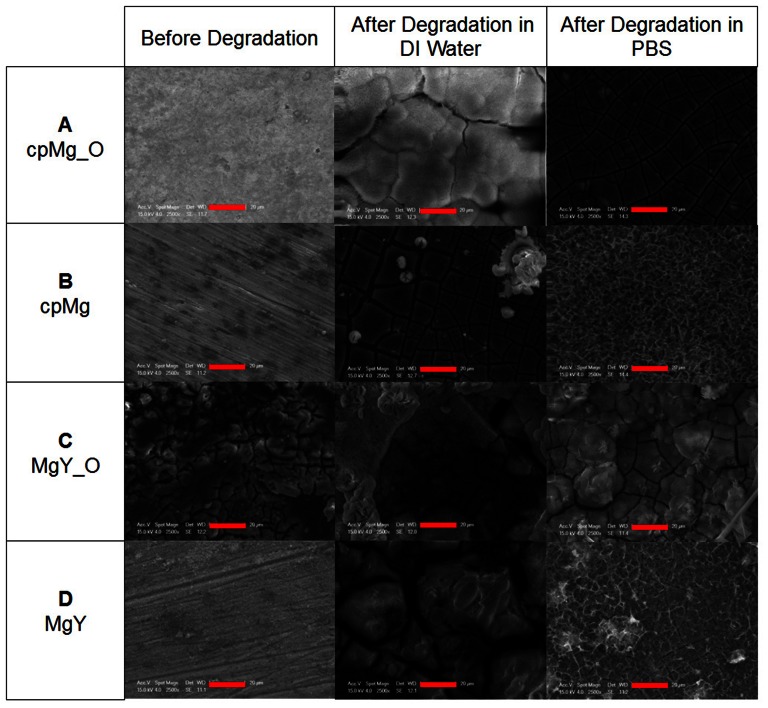
Surface microstructure of the (A, B) cpMg* and (C, D) MgY* samples before and after 24 hours of degradation in either DI water or PBS. Scale bars: 20 µm.

Scanning electron micrographs (Figure 9) showed that cpMg_O initially had a rough surface without cracks, MgY_O had a rough surface with cracks, and cpMg and MgY had relatively smooth surfaces with traces of scratch marks from grinding. Incubation in DI water and PBS both resulted in cracks and formation of degradation products on the surfaces of all samples. Incubation in PBS also caused formation of degradation products with a network-like morphology on the samples with metallic surfaces.

EDS analysis (Table 2) showed that alloy composition (MgY* versus cpMg*), initial surface condition (metallic versus oxide), and immersion solution (DI water versus PBS), all affected the surface composition after degradation. Degradation layers on the samples incorporated additional elements from PBS. All the cpMg* and MgY* samples incorporated more carbon (C) onto their surface in DI water than they did in PBS. Before degradation, the MgY_O samples had 5.20±0.71 wt.% Y on the surface and the MgY samples had 3.39±0.72 wt.% Y, both close to theoretical value of 4 wt.% Y. After degradation, Y percentage decreased on the surface of MgY_O samples in both DI water and PBS. In contrast, Y percentage increased on the surface of MgY in DI water.

**Table 2:** The surface elemental composition of the cpMg* and MgY* before and after 24 hours of degradation in DI water or PBS.

cpMg_O	Before degradation	Degradation in DI water	Degradation in PBS
Element	At.%	At.%	At.%
C	0	21.51±0.16	9.86±0.80
O	12.80±0.86	57.95±0.13	49.75±1.53
Na	0	0	8.78±1.03
Mg	87.19±0.86	20.52±0.03	21.28±1.43
P	0	0	9.93±0.39
Cl	0	0	0.38±0.66

cpMg	Before degradation	Degradation in DI water	Degradation in PBS
Element	At.%	At.%	At.%
C	0	20.09±1.60	12.57±0.43
O	10.96±2.42	49.54±3.08	53.18±0.97
Na	0	0	6.53±0.79
Mg	89.03±2.42	30.36±1.57	17.42±1.12
P	0	0	8.40±0.61
Cl	0	0	1.88±0.73

MgY_O	Before degradation	Degradation in DI water	Degradation in PBS
Element	At.%	At.%	At.%
C	16.56±3.05	12.72±1.96	10.45±9.07
O	55.63±1.53	58.27±0.94	55.81±4.85
Na	0	0	10.14±1.24
Mg	26.68±1.71	28.36±3.66	14.91±1.60
Y	1.11±0.13	0.63±0.44	0.35±0.05
P	0	0	8.11±1.13
Cl	0	0	0.07±0.06

MgY	Before degradation	Degradation in DI water	Degradation in PBS
Element	At.%	At.%	At.%
C	0	25.85±0.89	13.58±0.44
O	18.98±0.54	52.23±1.37	47.57±1.43
Na	0	0	11.28±0.59
Mg	80.12±0.37	19.78±1.58	18.36±0.68
Y	0.89±0.19	2.12±1.01	0.91±0.20
P	0	0	7.25±0.64
Cl	0	0	1.02±0.62

## Discussion

### The effects of surface and composition on the sample degradation in DI water

The results of this study confirmed that both surface (metallic versus oxide) and composition (alloying with Y versus pure Mg) contributed to the degradation in DI water. The surface effects were more pronounced for MgY* than for cpMg* samples in DI water, as demonstrated in Figures 2 and 3. The initial alkalinity in the DI water containing cpMg was caused by the degradation reactions. A protective thermal oxide layer was already present on cpMg_O, so reaction 1a was less prevalent initially. A more uniform degradation layer formed on cpMg* in DI water due to more uniform microstructure of pure Mg. As a result, free corrosion (general dissolution of Mg) rather than pitting corrosion was dominant for the cpMg* samples in DI water, as shown in Figure 2. In free corrosion mode, cpMg* degraded gradually without sudden release of sample fragments. The addition of Y as an alloying element accelerated the degradation in DI water. Yttrium had a net promoting effect on MgY* degradation in DI water due to instability of the degradation layers, as demonstrated by release of surface fragments from the samples. MgY_O degraded more slowly than MgY in DI water because the thermal oxide layer provided better protection as compared to the degradation layer formed by reaction 1.

### The effects of surface and composition on the sample degradation in PBS

It is still true that both surface (metallic versus oxide) and composition (alloying with Y versus pure Mg) contributed to the degradation in PBS. The surface effects were still more pronounced for MgY* than for cpMg* samples in PBS, as shown in Figures 5 and 6. In PBS, penetrating and undermining by Cl^−^ ions changed the degradation mode of cpMg* from free corrosion to localized corrosion. Cl^−^ attack also increased localized corrosion on MgY*. MgY was the only sample that lasted longer in PBS than in DI water, possibly because the effect of alloying with Y was more significant in PBS and a more stable degradation layer was able to form on MgY surface by incorporating salt ions from PBS.

**Figure 10:**
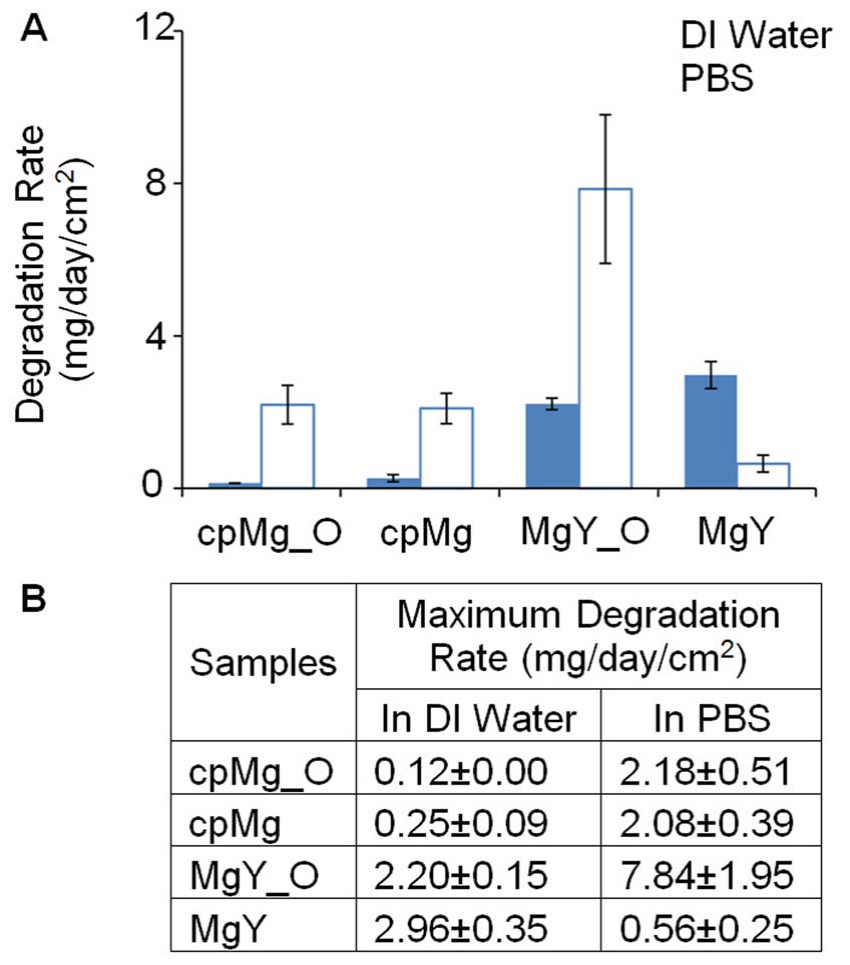
The maximum degradation rates of cpMg* and MgY* samples in DI water and in PBS. Data were calculated based on sample mass change using Eq 1. Values are average ± standard deviation; N = 3.

The importance of surface and composition, as well as presence of physiological salts on degradation was demonstrated in Figure 10. The maximum degradation rates of cpMg* and MgY* were calculated using the Equation 1 below based on the mass change data.
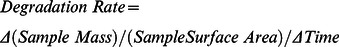
(Eq 1)


Considering that cpMg_O had a similar degradation rate as cpMg either in DI water or PBS, metallic versus oxide surface did not have significant effects on degradation of pure Mg. However, MgY degraded faster than MgY_O in DI water and vice versa in PBS. This indicated that surface condition (with or without oxide layer) and composition of immersion solution (with or without physiological ions) both had significant effects on the degradation. In other words, the protective surface barrier formed by Y passivation was influenced by both the initial surface microstructure and the composition of the immersion solution.

### Interacting factors that influenced cpMg* and MgY* degradation

Alloy composition, sample surface condition, and immersion media type significantly affected the sample degradation rates not only as factors acting separately but also as factors interacting with each other. The low *p* values (<0.05) for each individual factor, the two-way interactions, and the three-way interaction, as listed in Table 1, confirmed this.

The interaction plots demonstrated significant interactions between the three factors upon the sample lifetime (Figure 8). The almost parallel slopes in the left plot of Figure 8A suggested that the alloy-surface interaction was minor in DI water, while the “<” in the left plot showed the alloy-surface interaction was significant in PBS. The negative slopes in DI water indicated that the combination of MgY with either oxide or metallic surface decreased sample lifetime. Similarly, the negative slope for the oxide surface in PBS indicated that the combination of MgY with oxide surface decreased sample lifetime. In contrast, the positive slope for the metallic surface in PBS indicated that the combination of MgY and metallic surface prolonged sample lifetime.

In Figure 8B, the small angle between the slopes on the left plot indicated some observable alloy-media interaction for samples with oxide surface, and the negative slopes indicated that cpMg had a longer sample lifetime than MgY in either media when samples had oxide surfaces. The “X” shaped slopes on the right indicated significant interaction between alloy and media for samples with metallic surfaces. The “X” shape indicated that sample lifetime was longer for cpMg and shorter for MgY in DI water, but vice versa in PBS. This suggested that the degradation behavior of magnesium alloys could be reversed by different physiological conditions (e.g. concentration of salt ions). Because of this important implication, the design of a biodegradable metal must be tailored for specific anatomical locations or specific environmental conditions in the body.

In Figure 8C, the small angle between the slopes on the left plot indicated little media-surface interaction for cpMg* samples, and the positive slopes indicated that cpMg* had a longer lifetime in DI water than PBS. The larger angle between the slopes on the right plot indicated significant interaction between media and surface for MgY* samples. The “X” shape for the right plot showed that MgY_O had a shorter lifetime in PBS and a longer lifetime in DI water, but vice versa for MgY.

Understanding the interactions that control magnesium degradation is a crucial step in developing magnesium alloys as biodegradable implant materials. Physiological fluids are rich in aggressive ions that not only interact with alloy and surface directly, but also alter the effects of alloying and surface on degradation behavior. These interactions must be taken into account when designing biodegradable metallic implants.

### The effects of surface conditions on the degradation rate and mode

SEM images showed that the surfaces of cpMg_O, cpMg, and MgY were free of cracks initially, which limited penetration of aggressive Cl^−^ ions. In contrast, MgY_O had a significantly cracked surface initially, which increased its vulnerability to aggressive Cl^−^ ions. All cpMg* and MgY* samples had cracked surfaces after incubation in DI water. The thermal oxide layer on the MgY_O surface provided some initial protection from degradation in DI water, but the cracked surface exposed underneath metallic substrates to Cl^−^ ions. Electrochemical transport processes may have led to concentrated Cl^−^ ions in the cracks, which would cause severe undermining. Therefore, the thermal oxide surface protected MgY_O in the fluid lacking aggressive ions (i.e., DI water), but accelerated the degradation in the fluid containing aggressive ions (i.e., PBS). Severe undermining by Cl^−^ in PBS quickly caused the original thermal oxide layer breaking off from MgY_O. The loss of the oxide layer at some sites led to localized corrosion that continued to propagate. Continuous propagation of the localized corrosion sites may have prevented the formation or maintenance of a protective layer on the MgY_O surface. Thus, MgY_O degraded the fastest in PBS.

MgY and cpMg initially had metallic surface without surface oxide layer or cracks. Because of this, their degradation distributed across the entire sample surface rather than crack sites. Moreover, the initial degradation products formed a network-like morphology on MgY and cpMg in PBS (Figure 9). This network morphology of degradation products may have protected the surface underneath and physically restrained the release of large surface fragments, which limited the propagation of localized corrosion. As a result, a protective degradation layer was able to form on the metallic surfaces of MgY and cpMg and their degradation was slower than the respective samples with oxide surfaces in PBS. Eventually, MgY broke into fragments because the propagation of localized corrosion became too severe to keep the protective degradation layer intact.

Surface elemental composition played an important role in determining the susceptibility of samples to degradation. The amount of Y decreased on the surface of MgY_O after 24-h exposure to either immersion solution. MgY_O incubated in PBS had the lowest magnesium content on the surface and the greatest mass gain among all the samples. The low percentage of magnesium on the surface prevented the formation of an effective degradation layer in PBS. The absence of a stable surface layer compromised the protective effects of Y and other protective components like carbonate or phosphate. In contrast to MgY_O, the MgY surface became Y rich after degradation in DI water. MgY degraded the slowest in PBS because the degradation layer contained protective elements (e.g. carbonates) that might have provided a more stable protection than surface oxides. Because of the stable degradation layer, it took Cl^−^ ions longer to penetrate and undermine the surface. Eventually, the degradation layer was undermined so severely that it provided little protection. Therefore, after reaching the peak mass, the slope of MgY mass loss was similar as cpMg in PBS.

### Conclusions

This study demonstrated that the presence or absence of yttrium in magnesium alloys, the presence or absence of surface oxides, and the presence or absence of physiological ions in the immersion fluid collectively contributed to magnesium degradation, and interacted with one another on influencing magnesium degradation rate and mode. Specifically, Yttrium had a net degradation promoting effect for the MgY alloy in the DI water whether it had a metallic or oxide surfaces. However, in PBS, Y had a temporary net degradation inhibiting effect for the MgY alloy with the metallic surface, in contrast to a net degradation promoting effect for the same alloy with the oxide surface. Since physiological fluids in the human body contain aggressive ions such as Cl^−^, this study showed that commercially pure magnesium degraded faster in PBS than in DI water, while the MgY alloy with metallic surface degraded slower in PBS than in DI water. This study revealed the complex interrelationships of these factors and their respective contributions to magnesium degradation. The results of this study not only improved our understanding of magnesium degradation in a simulated physiological environment, but also presented the key factors to consider when designing next-generation biodegradable metallic implants and devices.
